# Case Report: Outpatient management of rectal infiltration by iodinated polyethylene glycol hydrogel and stabilized hyaluronic acid spacers in patients with prostate cancer

**DOI:** 10.3389/fonc.2026.1885291

**Published:** 2026-09-22

**Authors:** Jessica T. Lee, Brian C. T. Pien, Andy K. Chon, Robert F. Wong, John L. Lederer

**Affiliations:** 1 University of Hawaii Cancer Center, Honolulu, HI, United States; 2 Department of Medicine, John A. Burns School of Medicine, University of Hawaii, Honolulu, HI, United States; 3 Department of Radiology, Hawaii Pacific Health, Honolulu, HI, United States; 4 Pacific Endoscopy Center, Pearl City, HI, United States; 5 University of Hawaii Cancer Consortium, Honolulu, HI, United States; 6 Cancer Center of Hawaii, Honolulu, HI, United States

**Keywords:** hyaluronic acid, iodinated polyethylene glycol (PEG)-based hydrogel, outpatient management rectal infiltration, prostate cancer, radiation therapy

## Abstract

Prostate cancer is one of the most commonly diagnosed cancers in men worldwide. Radiation therapy is a widely used treatment for prostate cancer, but it carries the risk of adverse effects on adjacent organs such as the rectum. Therefore, to provide additional protection for the rectum during prostate radiation, perirectal spacers are inserted into Denonvilliers’ space, the thin fascial layer located behind the prostate gland and in front of the anterior rectal wall. Polyethylene glycol (PEG)-based hydrogels and recently FDA-approved stabilized hyaluronic acid (sHA) spacers have been designed to create a biodegradable space between the prostate and rectum, with each having its own unique characteristics and both having the potential to infiltrate the rectum. We present two clinical cases of rectal infiltration associated with either the insertion of a PEG-based hydrogel or an sHA spacer. Due to the distinct characteristics of the two spacer types, rectal infiltration was managed with either a conservative or definitive approach. With the PEG-based hydrogel, we implemented conservative management with antibiotics and close monitoring. In sharp contrast, for the sHA spacer, we proactively reversed the infiltration with hyaluronidase (Hyal) prior to starting radiation. These cases illustrate the clinical utility of these management approaches and contribute to the growing body of evidence on the management of potential complications associated with perirectal spacers, potentially supporting the use of newer spacer technologies.

## Introduction

Prostate cancer is one of the most frequently diagnosed cancers among men, with radiation therapy (RT) serving as a primary treatment modality for localized disease. However, because of the close anatomical proximity of the prostate to the rectum, the anterior rectal wall can be exposed to high doses of radiation, increasing the risk of gastrointestinal toxicities such as rectal pain, proctitis, bleeding, and long-term bowel dysfunction.

These risks are present in all fractionation schemes, including conventional fractionation (74–81 Gy delivered over 37–45 fractions over 7–8 weeks), moderate hypofractionation (60–72 Gy over 20–30 fractions delivered over 4–6 weeks), and ultrahypofractionation/Stereotactic Body Radiation Therapy SBRT regimens (36–42.7 Gy over 4–7 fractions over 2–3 weeks). While hypofractionation is convenient and cost-effective, studies suggest it may lead to a higher incidence of acute grade 2 or higher gastrointestinal (GI) toxicities (1–3). To mitigate these risks, perirectal spacers have been introduced to physically separate the rectum from the prostate, reducing radiation dose to the rectal wall and improving bowel-related quality of life (QOL). In the Hydrogel Spacer Pivotal trial, 222 prostate cancer patients were randomized to a polyethylene glycol (PEG) hydrogel spacer or no spacer (control) before undergoing conventional radiation. Patients who received the PEG hydrogel spacer had a significantly lower rate of late grade ≥ 2 rectal toxicity (2.0%) than those in the control group (7.0%), along with improved rectal dose reduction and bowel QOL (4). In the Barrigel^®^ Pivotal clinical trial, 201 patients with prostate cancer were randomly assigned to receive either a stabilized hyaluronic acid (sHA) rectal spacer or no spacer before undergoing moderate hypofractionation. Among patients who received an sHA spacer, 98.5% achieved a ≥ 25% reduction in the volume of the rectum receiving 54 Gy, and the rate of acute grade ≥ 2 GI toxicity was lower (2.9%) than that in the control group (13.8%) (5).

Perirectal spacers are implanted using a transperineal approach under transrectal ultrasound (TRUS) guidance, together with the placement of fiducial markers in the prostate. However, complications have been reported. If the rectal wall is inadvertently punctured or if the gel is misplaced, it may infiltrate or compress rectal tissue, potentially causing ischemia, pain, infection, abscess formation, or, in rare cases, death (6).

One critical limitation of PEG-based spacers is the inability to accelerate resorption. Once injected, the gel remains in Denonvilliers’ space for 3–6 months before being slowly broken down by hydrolysis and excreted via the kidneys (7). If complications arise, there is no rapid method to hydrolyze the gel, and management may require hospitalization and intravenous antibiotics. In contrast, sHA-based spacers offer a key advantage because they can be broken down by the enzyme hyaluronidase (Hyal), allowing for reversal in the event of a misplaced injection. HA is naturally broken down by the body’s endogenous Hyal (8) over time, but when injected with exogenous Hyal, the sHA spacer can be broken down within days. In this report, for which consent was obtained from both patients, we highlight the clinical feasibility of an office-based outpatient workflow to manage these distinct complications. Specifically, we demonstrate a successful outpatient sHA enzymatic reversal alongside the conservative outpatient management of rectal infiltration caused by an iodinated PEG (iPEG) hydrogel.

## Case 1

### Management of rectal infiltration by iPEG hydrogel

A 73-year-old man was diagnosed with high-risk clinical stage IIIA (cT2, cN0, cM0, PSA greater than 20, grade 4-Gleason score 4 + 4 = 8) prostate adenocarcinoma on 1 July 2021. The patient had an unremarkable past medical history, no history of anal-rectal surgery, fistulas, or abscesses, and no documented genetic predispositions, although his family history was notable for a younger brother diagnosed with prostate cancer. He elected to undergo combination therapy with low-dose-rate brachytherapy, using palladium-103 (Pd-103) seeds, followed by external beam radiation therapy (EBRT), planned for 45 Gy over 25 fractions. The patient received neoadjuvant androgen deprivation therapy (ADT) with the first of three 6-month depot leuprolide injections on 30 August 2021, 2 months prior to the upfront permanent Pd-103 prostate seed implant (PSI) on 3 November 2021 (**Table 1**).

**Table 1:** Timeline of events regarding spacer infiltration, treatment, and management.

Case Type	Time from diagnosis (days)	Event	Intervention/Outcome
Case 1 (iPEG)	0	Biopsy	Prostate cancer diagnosis
	61	Treatment initiation	6-month depot leuprolide injection
	125	LDR brachytherapy with iPEG	Prostate received 100 Gy Pd-103 MPD
	133	ER visit: urinary retention	Foley catheter insertion
	179	Radiation plan	Started pelvic radiation
	194	ER visit: perianal pain	Oral pain medicines
	194	Radiation treatment break	Stopped EBRT after 19.8 Gy (11 fractions)
	224	Recurrent pain	Oral antibiotics/suspected proctitis by RT or iPEG
	232	MRI pelvis	Revealed rectal fistula
	242	Continued ADT regimen	6-month depot leuprolide injection
	264	GI procedure: sigmoidoscopy	Revealed rectal wall defect
	354	Completed 4-month antibiotics	Persistent resolution of GI symptoms
	438	Continuing ADT regimen	6-month depot leuprolide injection
	448	Office-based CT scan	Confirmed disappearance of iPEG gel
	452	Urology office	Foley catheter removed
	552	MRI pelvis	Small focal anterior tear, without fluid collection
	559	Follow-up	Observation chosen; EBRT/ADT ceased
Case 2 (sHA)	0	Biopsy	Prostate cancer diagnosis
	85	Treatment initiation	6-month depot leuprolide injection
	93	Preparation for radiation	Barrigel and gold fiducial marker placement
	106	MRI pelvis	Identification of spacer infiltration
	162	Office-based procedure	sHA reversal of rectal infiltration with hyaluronidase
	174	MRI pelvis	Confirmed spacer infiltration resolution
	290	EBRT completed	50.4 Gy in 28 fractions to the pelvis
	309	Radiation course completed	Prostate received 100 Gy Pd-103 MPD

The implant dose to the prostate gland was 100 Gy Minimum Peripheral Dose (MPD), using a total of 64 Pd-103 seeds with an activity of 1.383 mCi per source, for a total activity of 88.51 mCi. Immediately following the placement of the Pd-103 seeds, he underwent insertion of an iPEG hydrogel (SpaceOAR VUE™). Due to urinary retention, the patient required Foley catheterization, with a postvoid residual volume greater than 150 cm^3^. The patient initially reported perianal pain, improving over the next month, but failed voiding trials at the 1-, 3-, and 5-week follow-up visits in the urology office.

The patient subsequently started EBRT on 27 December 2021. After reaching 19.8 Gy over 11 fractions on 11 January 2022, the patient presented to the emergency department with recurrent perianal pain. He was subsequently discharged with analgesics. Upon returning to the radiation department, the patient was placed on a 2-week treatment break, with improvement in symptoms; however, perianal pain recurred 5 days later, before radiation was restarted. Pelvic magnetic resonance imaging (MRI) was performed on 18 February 2022 to determine the etiology of the rectal pain and assess spacer placement. Inflammatory changes were observed within the pelvis, consistent with postradiation changes. There were also findings of cystitis and proctitis, likely related to treatment effects. A focal apparent 4 mm × 9 mm full-thickness transmural defect was identified in the wall of the anterior lower rectum, at the level of the mid gland to the apex of the prostate gland. This finding was highly suspicious for focal injury. A thin sinus tract, measuring 3 mm in diameter, possibly extended from this collection inferiorly through the mesorectal fascia to the level of the anus. No associated abscess was identified (**Figure 1**). Sigmoidoscopy on 25 March 2021 revealed a deep, cratered ulcer in the distal rectum. There was a deep 1–2-cm ulcer in the anterior rectum, 2 cm proximal to the dentate line. There was no bleeding or foreign material identified (**Figure 2**, top row).

**Figure 1:**
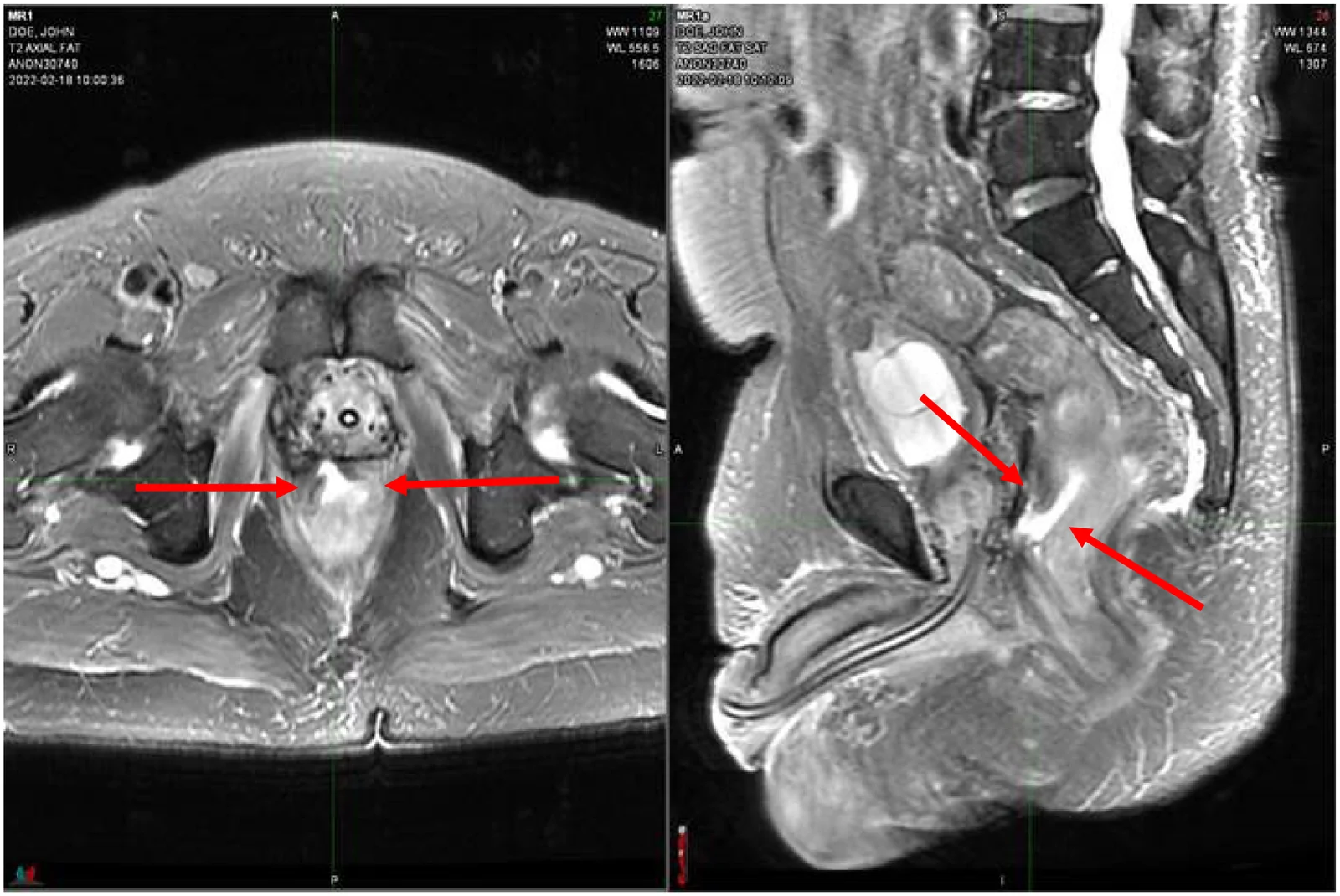
T2 axial (left) and T2 sagittal (right) images demonstrate a 4 mm × 9 mm full-thickness defect in the anterior rectal wall.

**Figure 2:**
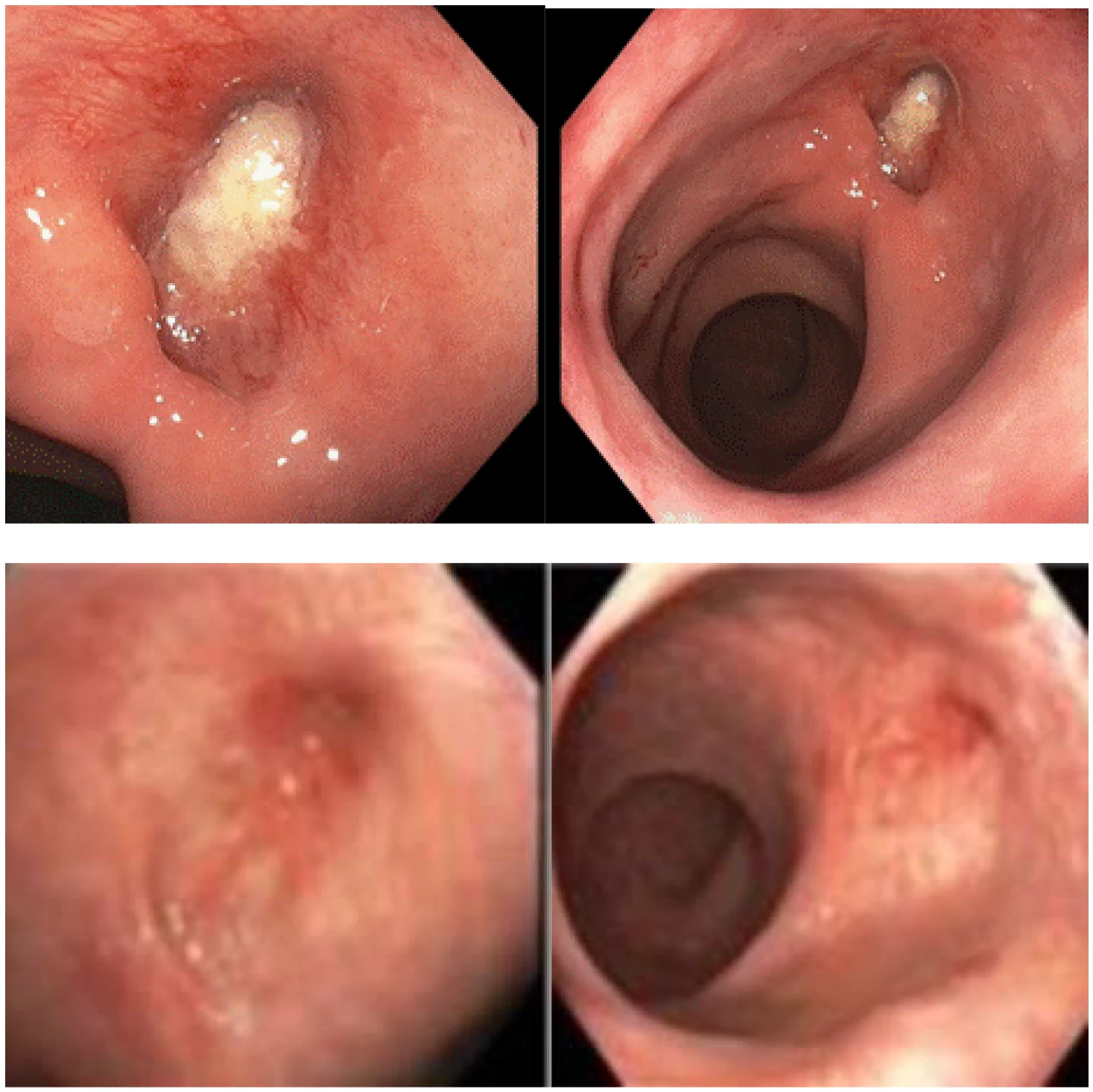
Top row: Sigmoidoscopy on 25 March 2022 identified a deep 1–2-cm ulcer in the anterior rectum, 2 cm proximal to the dentate line. Bottom row: Colonoscopy on 2 November 2022 demonstrated a mucosal defect in the distal rectum, compatible with a prior ulcer.

Based on these findings, both infectious disease and surgery were consulted. The surgical team recommended a colostomy to address the fistula, but the patient declined and opted for conservative management. Following infectious disease recommendations, the patient completed a 4-month course of ciprofloxacin (500 mg bid) and metronidazole (500 mg tid) from 10 February 2022 to 20 June 2022.

Upon completion of the antibiotics, serial departmental CT scans from 21 March 2022 to 12 September 2022 showed resolution of the fistula and near-complete disappearance of the iPEG hydrogel. Approximately 1 year later, colonoscopy on 2 November 2022 demonstrated a mucosal defect in the distal rectum, compatible with the prior ulcer, without evidence of a fistula or foreign body (**Figure 2**, bottom row). Pelvic MRI on 4 January 2023 showed prostate atrophy due to radiation therapy. The previously suspicious 2.7 cm × 1.4 cm × 1.8 cm mass in the anterior transition zone was no longer detected. There was evidence of a small focal anterior rectal tear, without an associated fluid collection, which had slightly decreased in prominence. The previously noted proctitis had completely resolved 9 months earlier.

On 11 January 2023, the patient returned for follow-up to review treatment options for his prostate cancer, including continuation of pelvic radiation therapy or observation. He opted for observation and received his third and last 45-mg leuprolide injection on 12 September 2022.

### Discussion

Iodinated PEG hydrogels are designed to protect the rectum by creating a physical barrier between the rectum and prostate, and were first made available in the USA in 2019 and released worldwide in November 2020 (9). The parent product of SpaceOAR Vue is SpaceOAR, and the Food and Drug Administration (FDA) issued *de novo* clearance for the SpaceOAR System on 1 April 2015 (10). The hydrogel is formed by mixing two solutions, including a trilysine buffer/PEG precursor and a salt buffer accelerant, via a Y-connector, and is then injected transperineally through an 18-gauge needle under TRUS guidance (11).

One of the primary advantages of iPEG is its visibility on CT imaging, which facilitates treatment planning and verification in centers without MRI access (12). However, despite this benefit, CT imaging alone cannot reliably detect rectal wall infiltration (RWI), limiting its ability to replace MRI when complications are suspected (13, 14).

In this case, the patient developed rectal symptoms that may represent local inflammation from both the transmural spacer infiltration and concurrent radiation-induced proctitis from the prostate seed implant. While concurrent radiation therapy is a confounding factor that makes it difficult to isolate the primary cause, management should still include empiric outpatient antibiotic treatment for potential infection. In contrast, Grossman et al. retrospectively reported no association between rectal wall injury and subsequent radiation. However, the authors acknowledged that the study was underpowered, with only seven patients having rectal wall injury from the clinical trial (15).

Reported technical issues with iPEG include premature polymerization within the applicator, preventing proper injection, and failure to form the hydrogel entirely (6, 16). These issues, combined with improper needle placement, the need for rapid delivery to avoid Y-connector clogging, and the inability to shape the space, may contribute to the inadvertent RWI. In 2022, the reported incidence of adverse events associated with a PEG spacer was found to be 0.0963% and was likely underreported (6, 17, 18). Although rare, there have been cases of spontaneous resolution of complications (19).

Case reports have described both inpatient and outpatient approaches to managing these complications. In one case, a patient with a rectal ulcer from spacer infiltration was managed conservatively with hospitalization, IV fluids, fasting, and blood transfusions to facilitate mucosal healing (19). Another report describes a symptomatic patient who experienced rectal bleeding and mucous discharge due to rectal penetration but spontaneously healed without surgical or inpatient intervention (20). Similarly, the patient in the present case was moderately symptomatic, with evidence of transmural infiltration by an iPEG spacer, and was managed conservatively and successfully treated with oral antibiotics.

## Case 2

### Management of rectal infiltration with stabilized hyaluronic acid

A 76-year-old man was diagnosed with unfavorable intermediate-risk prostate cancer, clinical stage IIC (cT2b, cN0, cM0, grade 3, with PSA less than 10), on 15 August 2023. His past medical, family, and psychosocial histories were unremarkable, with no known genetic variants. He elected to undergo combination therapy with upfront EBRT of 45 Gy in 25 fractions, followed by low-dose rate (LDR) brachytherapy boost with Pd-103 seeds (**Table 1**).

In preparation for radiation therapy, the patient underwent sHA spacer (Barrigel™, Teleflex, Pleasanton, CA, USA) placement, with gold fiducials also implanted in the prostate on 16 November 2023. The patient received prophylactic antibiotics, a Fleet enema, and local lidocaine. The patient was asymptomatic both intra- and postoperatively, without pain, fevers, chills, or issues with bowel movements. An MRI on 29 November 2023 (**Figure 3A**) was completed for preplanning and confirmation of spacer placement. Imaging identified T2-hyperintense material located between the posterior prostate gland and anterior rectal wall, compatible with the correctly placed portion of the sHA implant. There was a spherical 12-mm T2-hyperintense focus within the anterior rectal wall, likely related to sHA implant infiltration. No pelvic lymphadenopathy was identified. A total of 1 cm^3^ of the T2-hyperintense mass was measured within the lower rectum. The patient reported no adverse symptoms.

**Figure 3:**
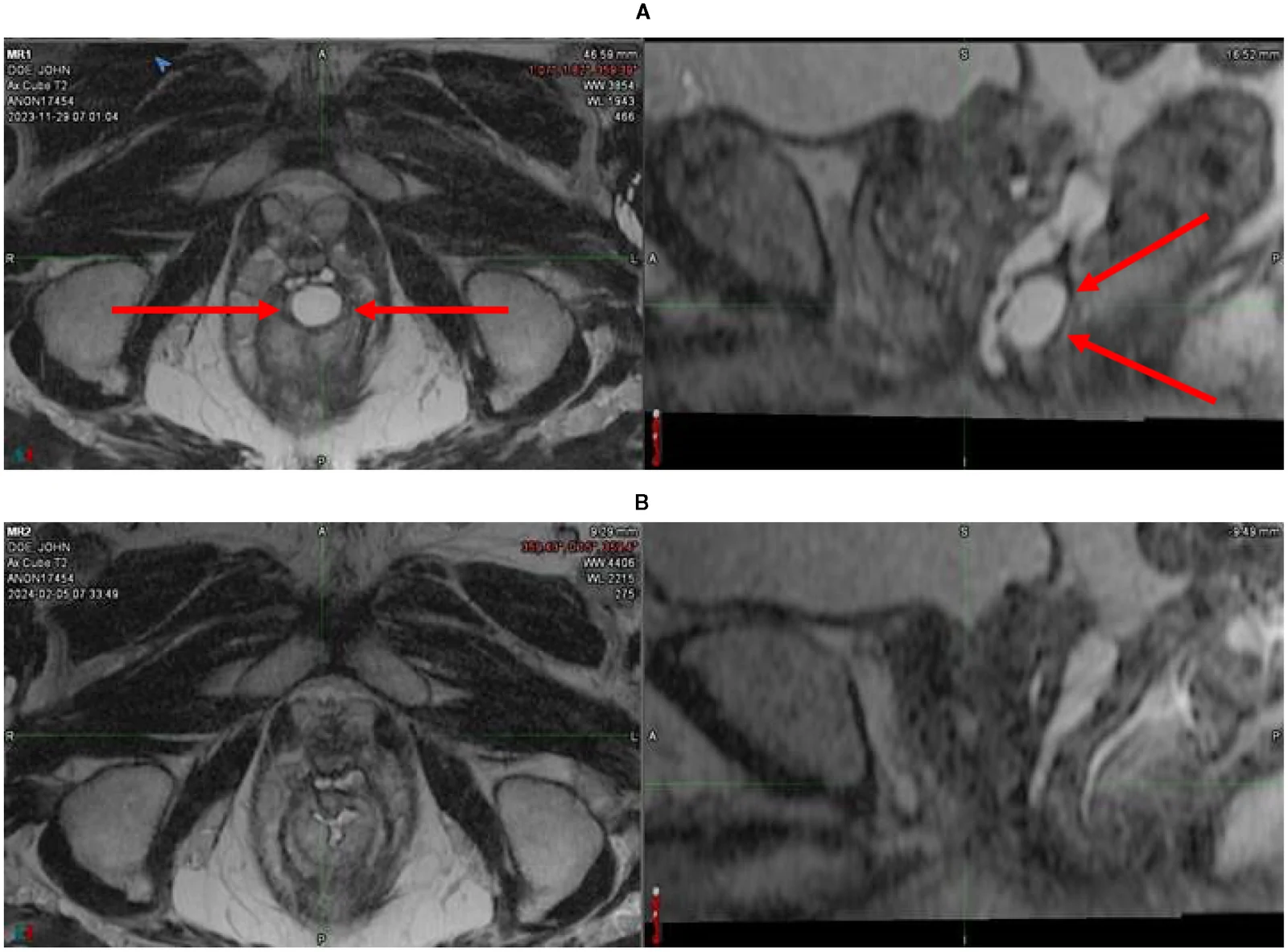
**(A)** (Top Row): Axial T2 (left); Sagittal T2 (right) - T2 hyperintense infiltration within the anterior rectal wall observed. **(B)** (Bottom Row): Axial T2 (left); Sagittal T2 (right) - T2 hyperintense material within the anterior rectal wall is no longer present.

Upon review, physician recommendation, and discussion, the patient opted to reverse the infiltration. On 24 January 2024, the patient underwent reversal of the 1-cm^3^ sHA misplacement in the outpatient office-based setting. Preparation with prophylactic antibiotics and local lidocaine for the Hyal injection was identical to that for the sHA spacer placement. We verified the infiltration by transrectal ultrasound. A total of 3 cm^3^ of Hyal at a concentration of 150 international units (IU)/cm^3^ was drawn into a 30-G spinal needle syringe, as the recommended amount of Hyal is 50 IU/0.1 cm^3^ of HA. With transrectal ultrasound guidance (**Figure 4**), Hyal was injected into the 1 cm^3^ of HA rectal infiltration. The patient had no pain or fever during or after the procedure.

**Figure 4:**
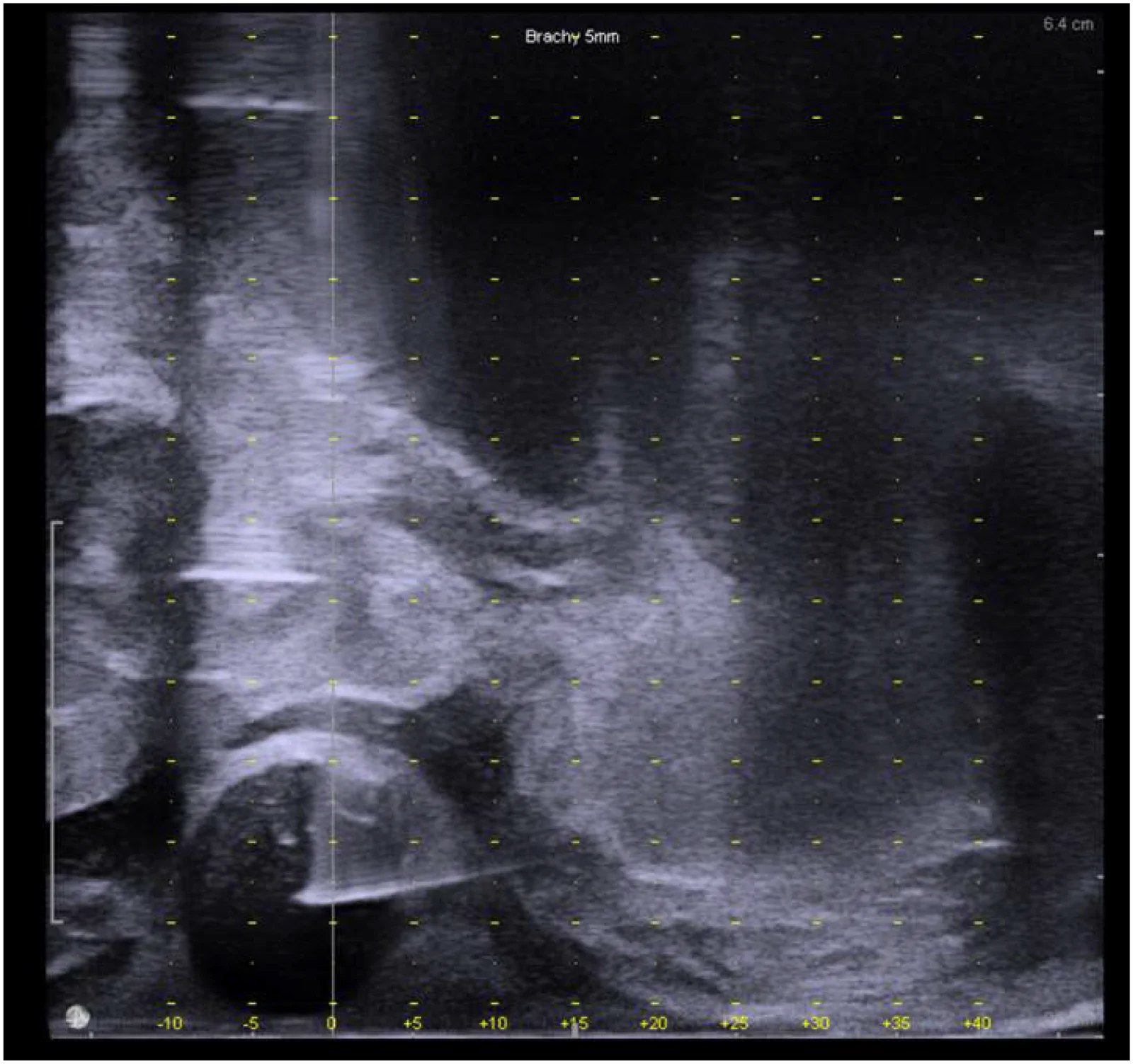
Transrectal ultrasound guidance showing HAS being injected into the 1 cm^3^ of HA rectal infiltration.

Repeat MRI on 5 February 2024 (**Figure 3B**) demonstrated T2-hyperintense material located between the posterior prostate gland and the anterior rectal wall, compatible with the correctly placed residual Barrigel implant, which measured up to 1.0 cm in AP dimension. The previously noted focus of T2 hyperintense material within the anterior rectal wall was no longer present. No pelvic lymphadenopathy was identified. Follow-up ultrasound on 14 February 2024 showed resolution of the RWI.

Following confirmation, the patient completed his EBRT on 31 May 2024, receiving 50.4 Gy in 28 fractions to the pelvic nodal regions, and then received a prostate implant with Pd-103 seeds to a 100-Gy MPD on 19 June 2024. There were no complications reported throughout his radiation course following HA placement and reversal.

### Discussion

The sHA spacer is a more recent development in rectal spacing technology for prostate cancer radiation therapy, having received FDA clearance in June 2022 (21). Unlike PEG hydrogels, which require the mixing of two components at the time of injection, the HA spacer is delivered in a premixed vial, enabling simpler application.

An advantage of sHA spacers lies in their ability to be sculpted, rather than relying on gel formation in Denonvilliers’ space. The nonpolymerizing property of sHA allows real-time adjustments during the procedure, offering flexibility for the physician to optimize placement.

One disadvantage of sHA spacers is the difficulty in visualizing them on CT scans. This may hinder postplacement imaging and verification. Furthermore, allergic reactions or delayed hypersensitivity responses to HA-based products have been reported in other settings, particularly in patients previously sensitized to Hyal (8).

RWI remains a potential risk despite improved delivery control. In a case report, a patient with a rectal infiltration raised concerns about tissue damage or complications during radiotherapy. The patient was asymptomatic but was admitted to the hospital to undergo sHA reversal with Hyal. The intervention was performed in an inpatient hospital setting, and the patient recovered without any complications (22). While complications can occur, sHA spacers offer the unique advantage of being reversible and potentially avoiding undesirable outcomes, as reported with misplaced hydrogel injections (23).

Understanding the depth and type of infiltration according to the Fischer–Valuck criteria may be helpful in guiding clinical decisions (25). Transmural infiltration, where the spacer penetrates all layers of the rectum, carries the highest risk for complications such as infection or radiation proctitis. Serosal and mucosal infiltrations, limited to the outermost and innermost layers, respectively, may carry lower risks, although they still require careful evaluation before proceeding with radiotherapy (15).

When deciding whether to proceed with radiation therapy following rectal infiltration by hydrogel spacers, careful consideration of the infiltration type and the patient’s symptoms is essential. Infiltration limited to the serosal or mucosal layers, particularly in asymptomatic patients with sHA volumes less than 1 cm^3^, may allow continuation of radiation therapy with close monitoring (**Figure 5**). This decision to continue may depend on the volume and extent of spacer involvement, although clear guidelines are still evolving. In fact, one review stated that there are insufficient data, as complications are rare (15, 24). In contrast, symptomatic patients, particularly those with transmural involvement, often require conservative management and close monitoring due to the risk of exacerbating complications with extreme perianal pain, ulceration, or abscess formation.

**Figure 5:**
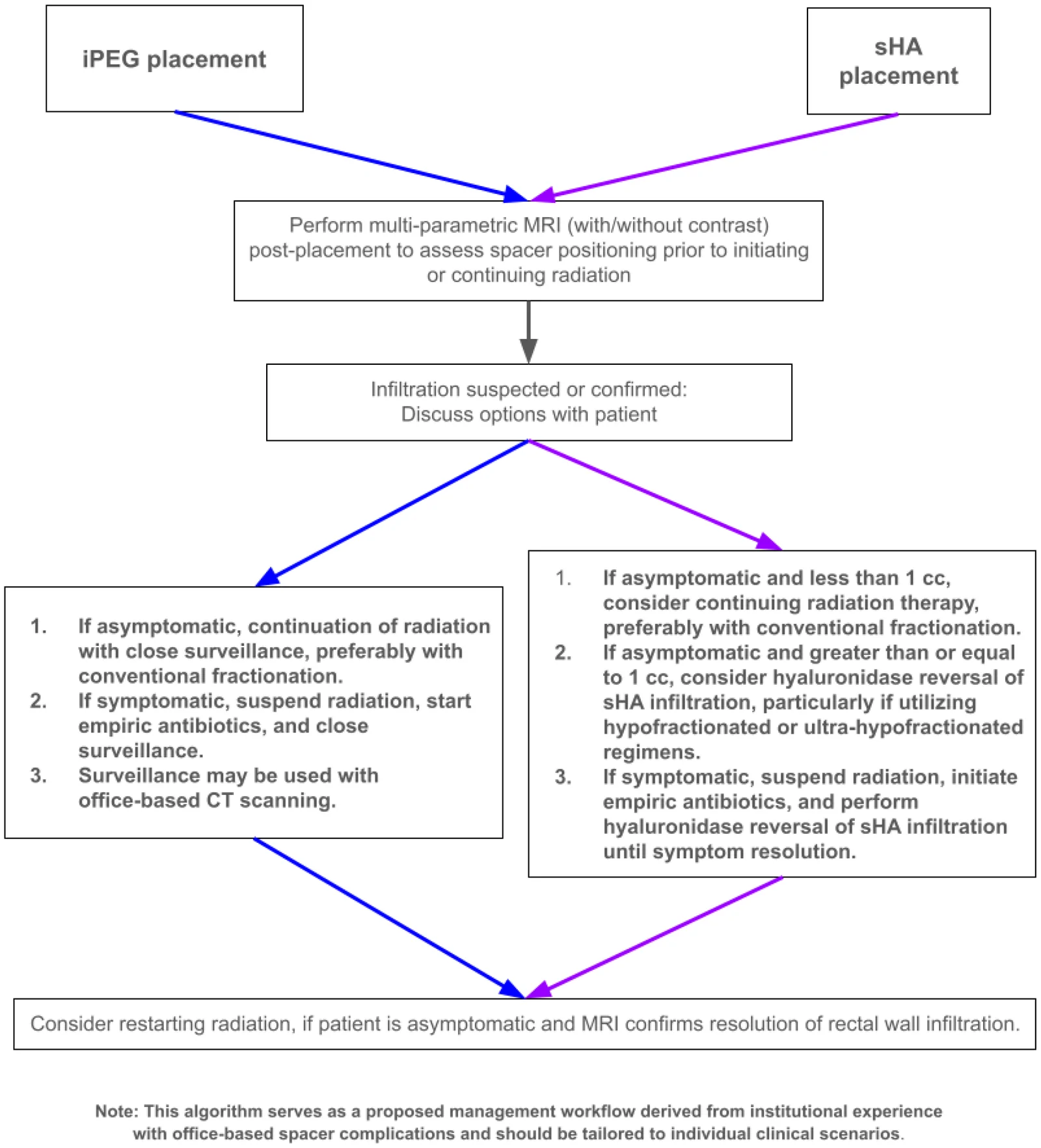
Proposed clinical decision-management algorithm for office-based hydrogel spacer complications.

As these infiltrative complications are rare, standardized clinical guidelines do not exist. To share our institutional workflow, we developed an office-based management algorithm (**Figure 5**). This framework is not intended to be universally applied or to imply that office-based care is mandatory for all patients. Instead, it demonstrates how outpatient surveillance and intervention can be safely utilized. All clinical paths must remain individualized based on spacer volume, patient symptoms, and individual risk profiles.

## Patient perspective

Both patients participated in shared clinical decision-making regarding the outpatient management of RWI and the treatment course. In case 1 (iPEG), the patient appreciated the conservative approach with home oral antibiotics and close monitoring in the office. He was relieved that aggressive management, such as a diverting colostomy, was avoided. In case 2 (sHA), the patient favored the office-based enzymatic reversal without anesthesia, which allowed him to complete radiation therapy with minimal disruption. They were satisfied with their prostate cancer control, confirmed by low to undetectable serial PSAs.

## Conclusion

Our findings align with previously reported literature, which demonstrated a similar or lower incidence of RWI: approximately two events in over 500 documented cases over eight years (6, 24, 25). These cases highlight the importance of MRI imaging in identifying patients with RWI, especially because CT imaging fails to reveal early or subtle changes, particularly in asymptomatic individuals. While randomized trials like PACE-B demonstrate comparable safety profiles between SBRT and conventional fractionation in the absence of procedural complications, those trials did not account for or include patients with rectal spacer infiltrations. The clinical consequences of delivering high-dose hypofractionated or SBRT regimens directly into a rectal wall compromised by misplaced biomaterials remain unknown and are potentially severe (23, 26–28).

Routine high-resolution MRI screening is helpful for detecting these infiltrations before treatment begins, allowing practitioners to modify their management strategy. Rather than defaulting to inpatient workflows, management should be personalized based on individual symptoms, depth of tissue involvement, and spacer biomaterial properties. Depending on the clinical presentation, this strategy may include switching to a conventional fractionation schedule to potentially decrease late complications, adopting a conservative observation period, or utilizing empiric oral antibiotics. Our institutional experience highlights that nonreversible iPEG hydrogels dictate vigilant conservative surveillance utilizing office-based CT scanning, while sHA spacers allow for safe, proactive enzymatic reversal using hyaluronidase within an office-based outpatient clinic setting.

## Data Availability

The original contributions presented in the study are included in the article/Supplementary Material. Further inquiries can be directed to the corresponding author.
