# Correction: Case Report: Outpatient management of rectal infiltration by iodinated polyethylene glycol hydrogel and stabilized hyaluronic acid spacers in patients with prostate cancer

**DOI:** 10.3389/fonc.2026.2020530

**Published:** 2026-10-20

**Authors:** Jessica T. Lee, Brian C. T. Pien, Andy K. Chon, Robert F. Wong, John L. Lederer

**Affiliations:** 1 University of Hawaii Cancer Center, Honolulu, HI, United States; 2 Department of Medicine, John A. Burns School of Medicine, University of Hawaii, Honolulu, HI, United States; 3 Department of Radiology, Hawaii Pacific Health, Honolulu, HI, United States; 4 Pacific Endoscopy Center, Pearl City, HI, United States; 5 University of Hawaii Cancer Consortium, Honolulu, HI, United States; 6 Cancer Center of Hawaii, Honolulu, HI, United States

**Keywords:** hyaluronic acid, iodinated polyethylene glycol (PEG)-based hydrogel, outpatient management rectal infiltration, prostate cancer, radiation therapy

Affiliation “University of Hawaii Cancer Center, Honolulu, HI, United States” was omitted from the paper. This affiliation has now been added for author Jessica T. Lee. Affiliation "Cancer Center of Hawaii, Honolulu, United States" was omitted from the paper. This affiliation has now been added to John L. Lederer.

Affiliation “St Francis Healthcare System of Hawaii, Honolulu, HI, United States” was erroneously included in the paper. This affiliation has now been removed for author Jessica T. Lee.

The original version of this article has been updated.

